# Spectrum of Genetic Variants Associated with Anterior Segment Dysgenesis in South Florida

**DOI:** 10.3390/genes11040350

**Published:** 2020-03-26

**Authors:** Saradadevi Thanikachalam, Elizabeth Hodapp, Ta C. Chang, Dayna Morel Swols, Filiz B. Cengiz, Shengru Guo, Mohammad F. Zafeer, Serhat Seyhan, Guney Bademci, William K. Scott, Alana Grajewski, Mustafa Tekin

**Affiliations:** 1 John P. Hussmann Institute for Human Genomics, University of Miami Miller School of Medicine, Miami, FL 33136, USA; saradadevi.thanikachalam@uth.tmc.edu (S.T.); fbsakc@yahoo.com (F.B.C.); sguo@med.miami.edu (S.G.); mxz625@med.miami.edu (M.F.Z.); drserhatseyhan@gmail.com (S.S.); g.bademci@med.miami.edu (G.B.); w.scott@med.miami.edu (W.K.S.); 2 Bascom Palmer Eye Institute, University of Miami Miller School of Medicine, Miami, FL 33136, USA; hodapp@med.miami.edu (E.H.); t.chang@med.miami.edu (T.C.C.); agrajewski@med.miami.edu (A.G.); 3 Department of Human Genetics, University of Miami Miller School of Medicine, Miami, FL 33136, USA; dmorel@med.miami.edu

**Keywords:** anterior segment dysgenesis, primary congenital glaucoma, exome sequencing

## Abstract

Anterior segment dysgenesis (ASD) comprises a wide spectrum of developmental conditions affecting the cornea, iris, and lens, which may be associated with abnormalities of other organs. To identify disease-causing variants, we performed exome sequencing in 24 South Florida families with ASD. We identified 12 likely causative variants in 10 families (42%), including single nucleotide or small insertion–deletion variants in B3GLCT, BMP4, CYP1B1, FOXC1, FOXE3, GJA1, PXDN, and TP63, and a large copy number variant involving PAX6. Four variants were novel. Each variant was detected only in one family. Likely causative variants were detected in 1 out of 7 black and 9 out of 17 white families. In conclusion, exome sequencing for ASD allows us to identify a wide spectrum of rare DNA variants in South Florida. Further studies will explore missing variants, especially in the black communities.

## 1. Introduction

Anterior segment dysgenesis (ASD) is a heterogeneous group of eye disorders affecting the cornea, iris, lens, zonule, trabecular meshwork, Schlemm canal, and ciliary body. Primary defects in migration or differentiation of the mesenchymal cells may cause ASD, and in turn impede aqueous humor outflow and elevate intraocular pressure [1]. Increased intraocular pressure is a major risk factor for glaucoma [2]. About 50% of individuals with ASD develop glaucoma, which often manifests before the age of 40 years [3,4].

ASD can present with ophthalmic findings only, such as in Peters anomaly and isolated aniridia, or as part of a multisystemic condition, such as Axenfeld–Rieger (AR; MIM 601542, 601090, 601499), Peters plus (MIM 261540), or SHORT (MIM 269880; short stature-hyperextensibility of joints or hernia or both-ocular depression-Rieger anomaly-teething delay) syndromes. Primary congenital glaucoma (PCG) is included in the ASD spectrum due to the presumed abnormal trabecular meshwork and Schlemm canal development [1,2].

In large families with multiple affected members, ASD is usually inherited as an autosomal dominant trait, though autosomal recessive inheritance has been reported [1]. Well-known ASD genes are CYP1B1 (MIM 601771), FOXC1 (MIM 601090), FOXE3 (MIM 601094), PAX6 (MIM 607108), and PITX2 (MIM 601542) [1]. Mutations in PAX6, PITX2, and FOXC1 do not always correlate with specific ASD phenotypes. Patients with AR syndrome and PCG may have FOXC1 mutations. PAX6 mutations can occur in both Peters anomaly and aniridia, and CYP1B1 mutations may be the cause of Peters anomaly and PCG. Phenotype or genotype alone is insufficiently precise to classify or diagnose ASD [2]. In the present study, we have investigated the genetic origin of isolated or syndromic ASD in the diverse population of South Florida.

## 2. Materials and Methods 

### 2.1. Subjects

We studied 24 (22 simplex and 2 multiplex) unrelated ASD-affected individuals (probands). Affected or unaffected family members were available for the study in 13 families. Families were recruited through the Bascom Palmer Eye Institute at the University of Miami, Miami, Florida. Probands were consecutive patients seen by an ophthalmologist for clinical diagnosis and management of ASD. Clinical evaluation of all affected individuals by an ophthalmologist included a slit lamp examination and dilated fundus exam. Further imaging and laboratory tests were performed when needed. DNA was extracted from peripheral leukocytes of each proband by standard protocols.

### 2.2. Genetic Screening 

We performed exome sequencing (ES) in the Hussman Institute for Human Genomics at the University of Miami. We used Agilent SureSelect Human All Exon 60 Mb V6 for in-solution enrichment of coding exons and flanking intronic sequences following the manufacturer’s standard protocol (Agilent). A HiSeq 3000 instrument (Illumina) was used for sequencing and Genome Analysis Toolkit software package used for variant calling [5,6]. During the analysis, we focused on specific genes with putative pathogenic variants previously found in individuals with ASD (Supplementary Materials Table S1).

We used Enlis genome software (https://www.enlis.com/) for annotation and variant filtering. As recommended, we filtered variants based on minor allele frequency of <0.005 in gnomAD (www.gnomad.broadinstitute.org) when considering a recessive mode of inheritance and <0.0005 when considering a dominant mode of inheritance [7,8]. Combined annotation dependent depletion (http://cadd.gs.washington.edu/) [9], MutationTaster (http://www.mutationtaster.org/) [10], and sorting intolerant from tolerant (http://sift.bii.a-star.edu.sg/) [11] in silico analysis tools were used for the pathogenicity prediction. Conservation of the variant was evaluated by using genomic evolutionary rate profiling (http://mendel.stanford.edu/SidowLab/downloads/gerp/) [12]. We used copy number inference from exome reads to detect copy number variants [13,14]. Sanger sequencing was performed to confirm the variants, and when other family members were available only those that showed complete segregation with the phenotype in the entire family were considered pathogenic. We used the American College of Medical Genetics guidelines to interpret variant pathogenicity [15,16].

## 3. Results

**Figure 1:**
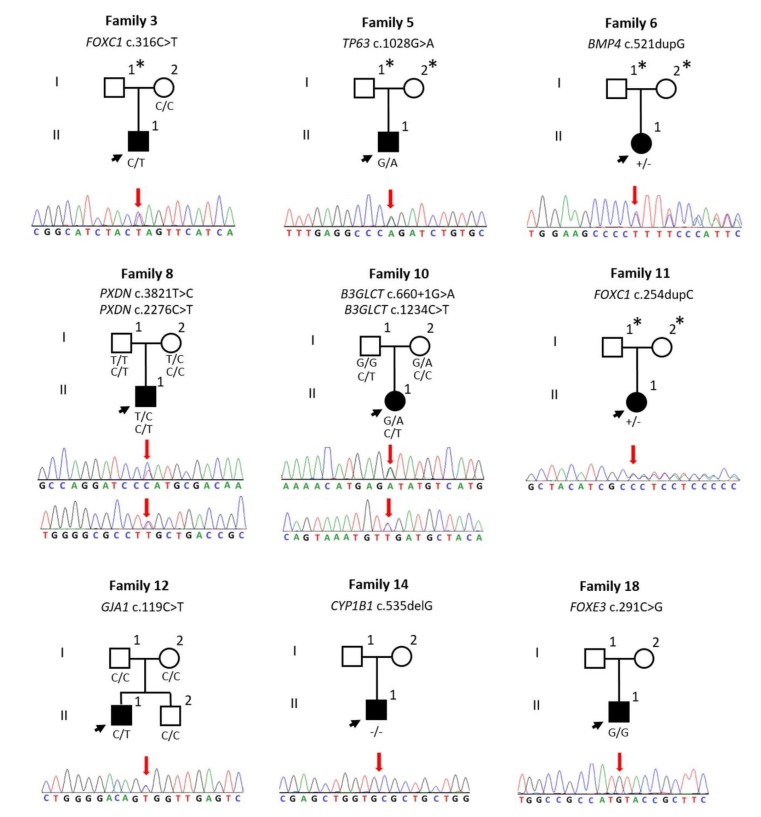
Pedigrees of the studied families, electropherograms, and segregation of the variants. Sanger sequencing traces represents identified variant positions (red arrow). * These individuals were not phenotypically evaluated therefore expected dominant transmission or de novo occurrence could not be demonstrated.

Based on the clinical evaluations, seven probands were considered to have syndromes associated with ASD (AR, Peters plus, and oculo–dento–digital syndromes (MIM 164200)), and 17 were diagnosed with isolated eye anomalies (Supplementary Materials Table S2). On average, each exome had 99.2%, 95.3%, and 89.4% of mappable bases of the Gencode defined exome represented by coverage of 1×, 5×, and 10× reads for ES, respectively. The average read depth was 71.9× and the coverage and average read depth are considered adequate for exome sequencing [17,18]. We detected nine pathogenic or likely pathogenic variants and three variants of uncertain significance (VUS) that potentially explain the observed phenotypes in 10 probands out of 24 (42%) (Figure 1, Table 1, Table 2, Supplementary Materials Figure S1 and Table S3 show phenotypic features of unsolved probands).

## 4. Discussion

In this study, we detected potentially causative variants in 42% of probands with ASD, which is higher than the reported proportion, which ranges from 10% to 25% [27]. Table 3 summarizes the characteristics of different genetic studies on ASD. Potential explanations for a higher detection rate of causative variants in our cohort are ethnicities studied, differences in case selection, the number of genes analyzed, and sample size. Our cohort consisted of a unique demographic from South Florida, including large Hispanic and Caribbean populations. Earlier studies focused on European, Asian, African, and Middle Eastern populations [27,28]. We did not identify recurrent variants enriched in our cohort; the difference between ethnicities of our cohort and those of earlier studies does not appear to explain our higher detection rate. In our cohort, families with Hispanic ancestry appear to have a higher detection rate (Hispanic vs. non-Hispanic is 7/12 vs. 3/12). Additionally, the difference between whites and blacks is noticeable: only 1 out of 7 black families is solved while 9 of 17 white families studied are found to have potentially causative variants. The majority of our black families were from the Caribbean, suggesting that the underlying genetic factors of ASD in the Caribbean remain largely unknown. Another important difference between our study and previous studies is the spectrum of ASD being analyzed. We looked at a wide range of ASD conditions, such as Peters anomaly, aniridia, AR syndrome, and PCG. Some other studies focused on a specific phenotype, such as Peters anomaly [23] or primary open-angle glaucoma/primary angle-closure glaucoma [27]. Recognized gene variants for some focused phenotypes are present in smaller portions of affected individuals, which likely contributes to higher detection rate in our study. We used ES to cover all genes previously associated with ASD and some previous studies used gene panels, which may not include all associated genes (Supplementary Materials Tables S1 and S4). While targeted next-generation sequencing gene panels potentially provide higher coverage for individual genes and lower cost, ES as a research tool reduces the need of development and validation of custom panels. Finally, our cohort is smaller in size compared to previous cohorts and may have a higher detection rate by chance. 

Identified variants in PAX6, FOXC1, TP63, BMP4, B3GLCT, and GJA1 are considered likely pathogenic or pathogenic based on American College of Medical Genetics (ACMG) guidelines. It should be noted that while GJA1 variant was de novo we did not confirm the parental origin. One proband with Peters anomaly was heterozygous for two VUSs in PXDN. One variant is a change from leucine to proline in position 1274. This variant affects a conserved residue and is predicted to affect protein function with a rare exome ensemble learner (REVEL) score of 0.776, which is a combination of 13 individual tools for pathogenicity prediction of missense variants [29]. The second variant shows a change from serine to leucine in position 759. This missense variant is also predicted to make an impact on protein function with a REVEL score of 0.8399. Each parent is heterozygous for one variant suggesting that these two variants are in trans (Figure 1). Biallelic PXDN variants have been reported with various eye anomalies including microphthalmia, congenital cataracts, microcornea, sclerocornea, and glaucoma [30,31]. Therefore, it is possible that the identified variants are the cause of Peters anomaly in our patient. Similarly, one proband was homozygous for the FOXE3 p.I97M variant, which is a VUS. The allele frequency of this variant on gnomAD is 0.00002015. Multiple in silico prediction tools show a damaging effect. This variant has been previously reported in a case with ASD [26]. Therefore, we consider the FOXE3 variant a likely cause of the eye phenotype in our proband. 

The observed phenotypes in the 10 probands and the variants identified are generally consistent with prior studies. However, in family five, the proband is heterozygous for a TP63 gene variant. Typically, TP63 mutations have been reported in Rapp–Hodgkin (MIM 129400), ADULT (acro–dermato–ungual–lacrimal–tooth; MIM 103285), EEC (ectrodactyly–ectodermal dysplasia–cleft lip/palate; MIM 604292), Hay–Wells (MIM 106260), limb–mammary (MIM 603543), and split hand/foot malformation (MIM 605289) syndromes. In addition to various systemic anomalies, eye findings of these syndromes include blue irides, photophobia, blepharophimosis, blepharitis, dacryocystitis, and lacrimal duct abnormalities [32,33,34,35,36,37]. Our proband was diagnosed with Peters anomaly in the right eye as well as with syndactyly of third and fourth toes in the left foot, vesicoureteral reflux, cleft lip and palate, possible glaucoma, and nasolacrimal abnormalities. All of these findings except for Peters anomaly have been reported in patients with TP63 variants. Another TP63 variant (p.R343W) affecting the same amino acid residue has been reported in a patient with glaucoma and decreased central corneal thickness as well as findings consistent with lacrimo–auriculo–dento–digital syndrome (MIM 149730) [38]. Peters anomaly in our patient and decreased corneal thickness associated with glaucoma in the previously reported patient may suggest that the Arg343 residue of TP63 plays a role in corneal development.

Some limitations of our study include the variety of ASD diagnoses among our patient sample. Our study encompasses patients with Peters anomaly, aniridia, AR syndrome, and PCG. Since the sample size is small for each ASD condition, it is difficult to assess the mutation detection rate in each ASD condition. Small family size and incomplete phenotypic evaluation of first-degree relatives made segregation studies difficult. Expected dominant transmission or de novo occurrence in families 3, 5, 6, and 11 could not be demonstrated due to unavailability of parental samples and lack of phenotypic evaluation of parents. Moreover, we did not identify a potentially causative variant in over half of our probands. With the available ES data and an increased cohort in the future, we expect to identify more variants to characterize the genetic features of ASD in South Florida. Finally, variants located in regulatory regions such as introns, promoters, and enhancers, may be studied with genome sequencing in the future.

## 5. Conclusions

We studied 24 families with ASD from South Florida and identified DNA variants potentially explaining 42% of our cohort. Further studies are required to compare different ASD demographics and identify underlying genetic variants on a larger scale.

## Figures and Tables

**Table 1:** Summary of the identified variants in this study.

Family ID	Gene	Transcript	Inh	Zyg	cDNA	Amino Acid Change	gnomAD	CADD	GERP RS	MutationTaster	SIFT	ACMG	ACMG Guidelines	Reference
1	*PAX6*	NM_000280.4	AD	HT	Large deletion (~266,752 bp)	Large deletion	N/A	N/A	N/A	N/A	N/A	LP	PVS1	Aradhya, 2012 [19]
3	*FOXC1*	NM_001453.2	AD	HT	c.316C>T	p.Q106*	N/A	38	3.8599	DC	N/A	P	PVS1, PM2, PP3, PP5	Dhaene, 2011 [20]
5	*TP63*	NM_003722.4	AD	HT	c.1028G>A	p.R343Q	N/A	33	5.8299	DC	DM	LP	PS3, PM2, PM5, PP3	Ianakiev, 2000 [21]
6	*BMP4*	NM_130851.3	AD	HT	c.521dupG	p.F175Lfs*8	N/A	35	5.1999	DC	N/A	P	PVS1, PM2, PP3	This study
8	*PXDN*	NM_012293.2	AR	HT	c.3821T>C	p.L1274P	N/A	25.8	5.4099	DC	DM	VUS	PM2, PP3	This study
				HT	c.2276C>T	p.S759L	0.00001204	32	5.63	DC	DM	VUS	PM2, PP3,	This study
10	*B3GLCT*	NM_194318.3	AR	HT	c.660+1G>A	Splice	0.0007602	34	6.0799	DC	N/A	P	PVS1, PP3, PP5	Lesnik Oberstein, 2006 [22]
				HT	c.1234C>T	p.R412*	N/A	36	3.23	DC	N/A	P	PVS1, PM2, PP3, PP5	Weh, 2014 [23]
11	*FOXC1*	NM_001453.2	AD	HT	c.254dupC	p.L86Afs*220	N/A	33	0.5139	DC	N/A	P	PVS1, PM1, PM2	This study
12	*GJA1*	NM_000165.4	AD	HT	c.119C>T	p.A40V	N/A	25.5	6.1599	DC	DM	P	PS3, PM1, PM2, PM6, PP2, PP3, PP5	Paznekas, 2003 [24]
14	*CYP1B1*	NM_000104.3	AR	HM	c.535delG	p.A179Rfs*18	0.00004797	24.2	2.5599	DC	N/A	P	PVS1, PM2, PP5	Belmouden, 2002 [25]
18	*FOXE3*	NM_012186.2	AR	HM	c.291C>G	p.I97M	0.00002015	22.4	1.1799	DC	DM	VUS	PM2, PP3	Quiroz-Casian, 2018 [26]

ACMG: American College of Medical Genetics guidelines, AD: autosomal dominant, AR: autosomal recessive, CADD: combined annotation dependent depletion, DC: disease-causing, DM: damaging, GERP RS: genomic evolutionary rate profiling rejected substitution, gnomAD: genome aggregation database, HM: homozygous, HT: heterozygous, Inh: inheritance, LP: likely pathogenic, N/A: not available, P: pathogenic, SIFT: sorting intolerant from tolerant, VUS: variants of uncertain significance, Zyg: zygosity.

**Table 2:** Phenotypic features of probands with causative variants.

Family-Individual ID	Sex	Simplex/Multiplex	Age (Years)	Ethnicity	Eye Phenotype	Additional Clinical Features	Gene
1-II:1	M	Sx	9	Black, non-Hispanic	Aniridia with glaucoma	-	*PAX6*
3-II:1	M	Sx	11	White, non-Hispanic	AR with glaucoma	-	*FOXC1*
5-II:1	M	Sx	9	White, Hispanic	Peters anomaly OD	Syndactyly of third and fourth toes in the left foot, vesicoureteral reflux, cleft lip and palate, and nasolacrimal abnormalities	*TP63*
6-II:1	F	Sx	9	White, Hispanic	Peters anomaly OU	-	*BMP4*
8-II:1	M	Sx	8	White, non-Hispanic	Peters anomaly OU	-	*PXDN*
10-II:1	F	Sx	8	White, Hispanic	Peters anomaly OU	-	*B3GLCT*
11-II:1	F	Sx	37	White, Hispanic	AR with glaucoma	-	*FOXC1*
12-II:1	M	Sx	13	White, Hispanic	Microphthalmia with glaucoma	Microdontia, underdeveloped alae nasi, syndactyly	*GJA1*
14-II:1	M	Sx	13	White, Hispanic	Peters anomaly OU	-	*CYP1B1*
18-II:1	M	Sx	6	White, Hispanic	Peters anomaly OU	-	*FOXE3*

AR: Axenfeld–Rieger anomaly, ASD: anterior segment dysgenesis, F: female, M: male, OD: right eye, OS: left eye, OU: both eyes, Sx: simplex.

**Table 3:** Causative variant detection in published studies.

Studies	Sample Size	Phenotypes	Population Studied	ES or Gene Panel	Causative Variants Detected in ASD
Weh et al. [23]	27	Syndromic Peters anomaly: 20 Isolated Peters anomaly: 7	Children’s Hospital of Wisconsin (USA) Population subtypes were not mentioned	ES	22.2% overall
Huang et al. [27]	257	POAG: 125 PACG: 132	Chinese: 257	ES of 43 genes associated with ASD, microcornea or microphthalmia	10.9% overall POAG: 8.80% PACG: 12.9%
Patel et al. [28]	277	MAC: 98 cases ASDA: 113 cases Other or syndromic: 8 cases RET: 49 cases Congenital cataracts and lens-associated conditions: 9 cases	White European: 139 South Asian: 21 Black African: 7 Arabic or Middle Eastern: 5 Black Caribbean: 2 Unknown: 91 Mixed/unclassified: 12	Oculome panel of 429 known eye disease genes	24.5% overall Congenital cataracts and lens-associated conditions: 88.9% RET: 42.8% Other or syndromic: 37.5% ASD: 24.8%
This study	24	Peters anomaly: 8 PCG: 6 AR: 5 Aniridia: 2 Congenital corneal dystrophy: 1 Microphthalmia with glaucoma: 1	White, Hispanic: 11 (7 solved) Black, Hispanic: 1 (0 solved) White, non-Hispanic: 6 (2 solved) Black, non-Hispanic: 6 (1 solved)	ES of 92 genes associated with eye phenotypes	42% overall Peters anomaly: 75% Aniridia: 50% Others: 50% AR: 40%

ASD: anterior segment dysgenesis, ASDA: anterior segment developmental anomalies including glaucoma, ES: exome sequencing, MAC: microphthalmia–anophthalmia–coloboma, PACG: primary angle-closure glaucoma, POAG: primary open-angle glaucoma, RET: retinal dystrophies.

## Supplementary Materials

The following are available online at https://www.mdpi.com/2073-4425/11/4/350/s1, Table S1: List of the genes used for the filtering by using ES in our cohort. Table S2: Syndromic and isolated subjects in both solved and unsolved probands. Table S3: Phenotypic features of the unsolved probands. Table S4: Characteristics of common genes for anterior segment dysgenesis. Figure S1: Representation of the PAX6 gene deletion by using CoNiFER.
