# Supplementary material for: Spectrum of Genetic Variants Associated with Anterior Segment Dysgenesis in South Florida
Source: Genes (Basel). 2020 Mar 26;11(4):350. doi: 10.3390/genes11040350 (PMC7230952; doi:10.3390/genes11040350)
Supplement: Supplementary file 1 [file genes-11-00350-s001.pdf]

Supplementary Information

Supplementary Table S1: List of the genes used for the filtering by using Exome Sequencing in our cohort.

ADAMTS10 (MIM 608990), *ADAMTS17* (MIM 607511), *ADAMTS18* (MIM 607512), *ALDH1A3* (MIM 600463), *ALDH18A1* (MIM 138250), *ANTXR1* (MIM 606410), *ASB10* (MIM 615054), *ATOH7* (MIM 609875), *B3GLCT* (MIM 610308), *BCOR (MIM 300485), BEST1* (MIM 607854), *BMP4 (MIM 112262), CHRDL1* (MIM 300350), *CHST6* (MIM 605294), *COL18A1* (MIM 120328), *COL2A1* (MIM 120140), *COL4A1* (MIM 120130), *COL8A2* (MIM 120252), *CPAMD8* (MIM 608841), *CREBBP* (MIM 600140), *CYP1B1* (MIM 601771), *DCN* (MIM 125255), *DDX58* (MIM 609631), *ELP4* (MIM 606985), *EYA1* (MIM 601653), *FBN1* (MIM 134797), *FLNA* (MIM 300017), *FOXC1* (MIM 601090), *FOXE3 (MIM 601094), FOXL2* (MIM 605597), *GDF6 (MIM 601147), GJA1* (MIM 121014), *HCCS* (MIM 300056), *IFIH1* (MIM 606951), *JAG1* (MIM 601920), *KERA* (MIM 603288), *KRT12* (MIM 601687), *KRT3* (MIM 148043), *LAMB2* (MIM 150325), *LMX1B* (MIM 602575), *LTBP2* (MIM 602091), *MAB21L2 (MIM 604357), MAF* (MIM 177075), *MFRP (MIM 606227), MYOC* (MIM 601652), *NOTCH2* (MIM 600275), *NTF4* (MIM 162662), *OPA1* (MIM 605290), *OPA3* (MIM 606580), *OPTN* (MIM 602432), *OTX2 (MIM 600037), OVOL2* (MIM 616441), *PAX2 (MIM 167409), PAX3* (MIM 606597), *PAX6* (MIM 607108), *PEX2* (MIM 170993), *PIKFYVE* (MIM 609414), *PIK3R1* (MIM 269880), *PITX2* (MIM 601542), *PITX3* (MIM 602669), *POMT1* (MIM 607423), *PRDM5* (MIM 614161), *PRSS56 (MIM 613858), PXDN (MIM 605158), RAB18* (MIM 602207), *RAB3GAP1* (MIM 602536), *RAB3GAP2* (MIM 609275), *RARB (MIM 180220), RAX (MIM 601881), SBF2* (MIM 607697), *SEC23A* (MIM 610511), *SH3PXD2B* (MIM 613293), *SHH (MIM 600725), SIX3* (MIM 603714), *SIX6* (MIM 606326), *SLC16A12* (MIM 611910), *SLC38A8* (MIM 615585), *SLC4A11* (MIM 610206), *SLC4A4* (MIM 603345), *SOX2 (MIM 184429), STRA6 (MIM 610745), TACSTD2* (MIM 137290), *TBC1D20* (MIM 611663), *TEK* (MIM 600221), *TGFBI* (MIM 601692), *TMEM98* (MIM 615949), *UBIAD1* (MIM 611632), *VCAN* (MIM 118661), *VSX1* (MIM 605020), *VSX2 (MIM 142993), WDR36* (MIM 609669), *ZEB1* (MIM 189909)

Supplementary Table S2: Syndromic and isolated subjects in both solved and unsolved probands.

Type of Probands	Syndromic	Isolated	Solve percentage
Solved Probands	4 (1 oculo-dental-digital syndrome, 2 Axenfeld Rieger syndrome, 1 Peters plus syndrome)	6	40% solved
Unsolved Probands	3 (3 Axenfeld Rieger syndrome)	11	21.4% solved

Supplementary Table S3: Phenotypic features of the unsolved probands.

Family-individual ID	Sex	Simplex/ Multiplex	Ethnicity	Eye Phenotype	Additional Clinical and Family Characteristics
2-II:1	M	Sx	Black, non-Hispanic	AR with glaucoma	-
4-II:1	M	Sx	White, Hispanic	Peters OD, anopthalmia OS	-
7-II:1	F	Sx	White, non-Hispanic	AR OD, corneal opacity OS	-
9-II:1	M	Mx	White, Hispanic	AR with glaucoma	Sister had PCG and father had glaucoma
13-II:1	M	Sx	Black, Hispanic	Aniridia	-
15-II:1	F	Sx	White, non-Hispanic	PCG	-
16-II:1	F	Sx	White, Hispanic	corneal dystrophy with suspected glaucoma	-
17:II-1	F	Sx	White, non-Hispanic	Peters anomaly OU	-
19-II:1	M	Sx	Black, non-Hispanic	PCG	-
20-II:1	F	Sx	White, non-Hispanic	PCG	-
21-II:1	M	Sx	Black, non-Hispanic	PCG	-
22-II:1	F	Sx	Black, non-Hispanic	PCG	-
23-II:1	M	Sx	White, Hispanic	PCG	-
24-II:1	M	Mx	Black, non-Hispanic	Congenital glaucoma	One sibling has glaucoma

M: male, **F:** female, **Sx:** simplex, **Mx:** multiplex, **y:** years, **AR:** Axenfeld Rieger anomaly, **OD:** Right eye, **OS**: Left eye

Supplementary Table S4: Characteristics of common genes for anterior segment dysgenesis

Gene	OMIM	Phenotype	Inheritance	Reported type of variants			
				Missense	Loss of function	CNVs	splice
CYP1B1	*617315*	Peters anomaly, corneal edema, corneal leucoma	AR	+	+	+	+
FOXC1	*601631*	Goniodysgenesis, Rieger anomaly, Peters anomaly, corectopia	AD	+	+	+	+
FOXE3	*610256*	Microphthalmia, sclerocornea, Peters anomaly	AR	+	+	NA	NA
PAX6	*604229*	Aniridia, Peters anomaly, keratitis, foveal hypoplasia, congenital cataract	AD	+	+	+	+
PITX2	*137600*	Glaucoma, hypoplastic iris stroma, goniodysgenesis	AD	+	+	+	+
PITX3	*107250*	Microcornea, synechiae, Peters anomaly	AD	+	+	NA	NA
PXDN	*269400*	Microcornea, iridocorneal dysgenesis, sclerocornea, cataract	AR	+	+	+	+

AD: autosomal dominant; **AR:** autosomal recessive; **Loss of function:** nonsense and frameshifts; **CNVs**: copy number variants

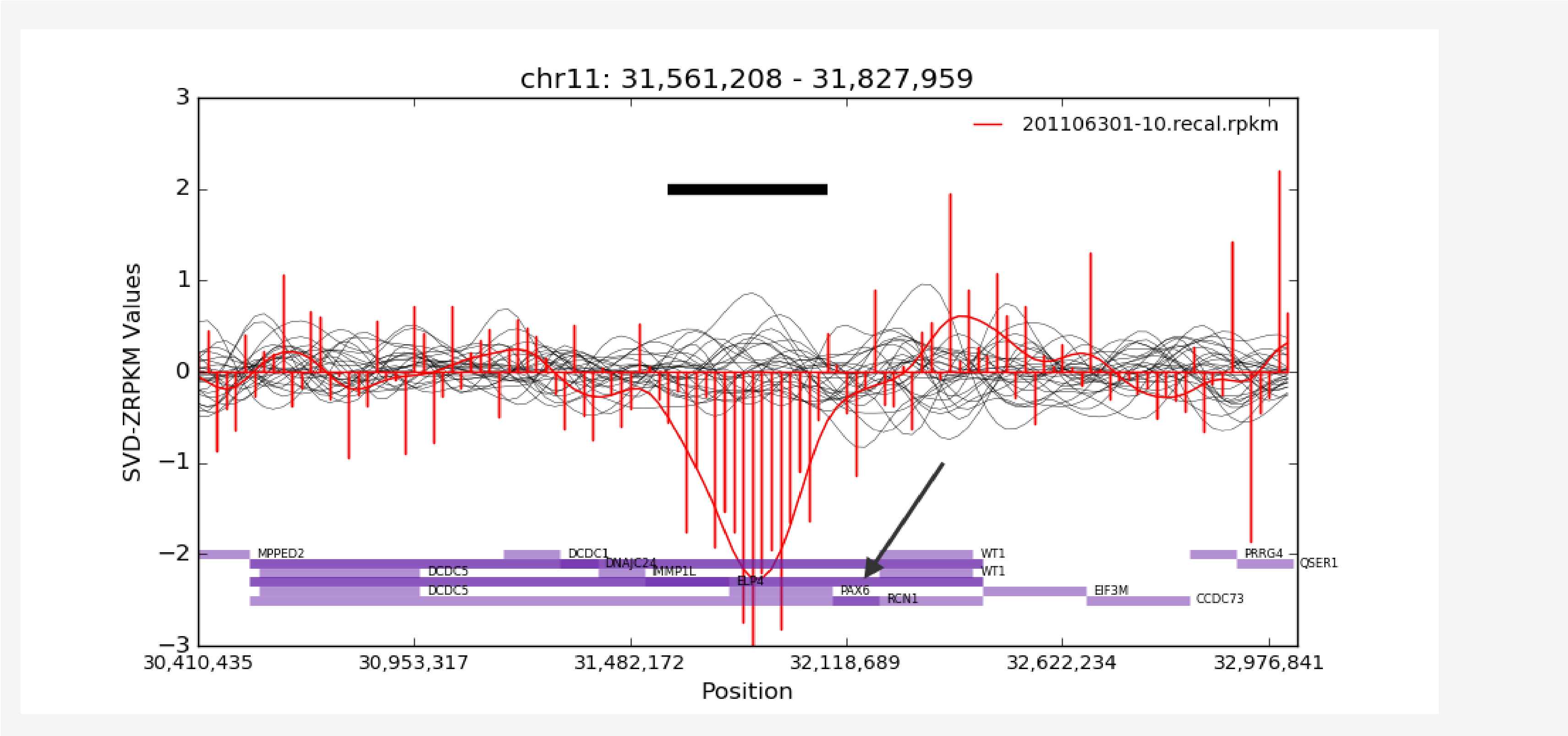

Supplementary Figure S1: Representation of the *PAX6* gene deletion by using CoNiFER. Black line shows *PAX6* gene region and black arrow shows *PAX6*. Coordinates are based on hg19.
